# Vaginal birth after cesarean section and its associated factors in Ethiopia: a systematic review and meta-analysis

**DOI:** 10.1038/s41598-023-34856-8

**Published:** 2023-05-15

**Authors:** Dagne Addisu, Natnael Atnafu Gebeyehu, Shimeles Biru, Yismaw Yimam Belachew

**Affiliations:** 1 Department of Midwifery, College of Health Sciences, Debre Tabor University, Debre Tabor, Ethiopia; 2 School of Midwifery, College of Health Science and Medicine, Wolaita Sodo University, Wolaita Sodo, Ethiopia; 3 Department of Obstetrics and Gynecology, School of Medicine, College of Health Sciences, Debre Tabor University, Debre Tabor, Ethiopia

**Keywords:** Health care, Medical research, Risk factors

## Abstract

The prevalence of cesarean sections is rising rapidly and is becoming a global issue. Vaginal birth after a cesarean section is one of the safest strategies that can be used to decrease the cesarean section rate. Different fragmented primary studies were done on the success rate of vaginal birth after cesarean section and its associated factors in Ethiopia. However, the findings were controversial and inconclusive. Therefore, this meta-analysis was intended to estimate the pooled success rate of vaginal birth after cesarean section and its associated factors in Ethiopia. Pertinent studies were searched in PubMed, Google Scholar, ScienceDirect, direct open-access journals, and Ethiopian universities' institutional repositories. The data were analyzed using Stata 17. The Newcastle–Ottawa quality assessment tool was used to assess the quality of the studies. I squared statistics and Egger’s regression tests were used to assess heterogeneity and publication bias, respectively. A random effects model was selected to estimate the pooled success rate of vaginal birth after cesarean section and its associated factors. The PROSPERO registration number for this review is CRD42023413715. A total of 10 studies were included. The pooled success rate of vaginal birth after a cesarean section was found to be 48.42%. Age less than 30 years (pooled odds ratio (OR) 3.75, 95% CI 1.92, 7.33), previous history of vaginal birth (OR 3.65, 95% CI 2.64, 504), ruptured amniotic membrane at admission (OR 2.87, 95% CI 1.94, 4.26), 4 cm or more cervical dilatation at admission (OR 4, 95% CI 2.33, 6.8), a low station at admission (OR 5.07, 95% CI 2.08, 12.34), and no history of stillbirth (OR 4.93, 95% CI 1.82, 13.36) were significantly associated with successful vaginal birth after cesarean section. In conclusion, the pooled success rate of vaginal birth after a cesarean section was low in Ethiopia. Therefore, the Ministry of Health should consider those identified factors and revise the management guidelines and eligibility criteria for a trial of labor after a cesarean section.

## Introduction

Worldwide, the cesarean section (CS) rate has increased dramatically and has become an international concern^1–4^. In 2018, the overall CS rate was 21.1% in the world, 25.7% in Europe, 23.1% in Asia, 42.8% in Latin America and the Caribbean, and 9.2% in Africa^3^.

The CS rate was 29.5% in Ethiopia^5^. According to a meta-analysis study conducted in Ethiopia, CS has been associated with a high rate of maternal and neonatal morbidity and mortality, such as postpartum hemorrhage, severe anemia, surgical site infection, maternal mortality, perinatal asphyxia, neonatal sepsis, and early neonatal death^5^.

Various factors are contributing to the increasing cesarean section rates. Women's and families' preferences, health professionals' views and beliefs, and healthcare organizations and financial structures are some of the factors contributing to the rising CS rate^3,4^. In addition, the shifts in the obstetrics field, such as the decreased use of operative vaginal delivery, the decline in vaginal breech delivery, and the decreased use of vaginal birth after cesarean (VBAC), have increased the global CS rates^1,5,6^.

Several attempts have been made to reduce the CS rate^1,7^. VBAC is one of the best strategies that can be used to decrease the CS rate and is associated with a lower incidence of maternal and neonatal morbidity and mortality as compared to repeat CS^2,8–12^. A successful trial of labor after cesarean (TOLAC) reduces the risk of blood loss, hysterectomy, and associated puerperal infections^13^. However, unsuccessful TOLAC increases the above-mentioned maternal morbidity, uterine rupture, and adverse perinatal outcomes^14–16^.

The success rates of VBAC vary widely in different countries, with a global success rate ranging from 60 to 80%^17,18^. It was around 80.7% in Taiwan^19^, 63.4% in the UK^20^, 73% in Iraq^1^, 72.1% in Pakistan^13^, 57.6% in the DRC^21^, and 61.7% in Nigeria^6^.

The success rate of VBAC is influenced by several factors. Some of the factors were the service provider's choice, cervical dilatation, prior vaginal delivery, younger maternal age, indication for previous CS, fetal weight, obesity, diabetes, gestational age, and hypertensive disorders complicating pregnancy^14,16,17,21–23^.

Although different fragmented primary studies were done in different district areas of Ethiopia, the overall success rate of VBAC is unknown. In addition, the success rate of VBAC obtained from those primary studies was widely variable, ranging from 35.07 to 69.4%^24,25^. Furthermore, the associated factors for VBAC found in those studies were controversial and inconclusive. Therefore, this meta-analysis was conducted to estimate the pooled success rate of VBAC and its associated factors in Ethiopia. The findings of this study may enable policymakers to design strategies for improving the success rate of vaginal birth after a cesarean section. The results of this study may also help obstetric care providers by enabling them to deliver evidence-based counseling on VBAC, which has a major impact on averting repeated CS.

## Methods

### Sources of information and search strategies

This review was carried out following the PRISMA (Preferred Reporting Items for Systematic Reviews and Meta-analyses) guideline^26^ (Table S1). The protocol was registered in PROSPERO. The PROSPERO registration number for this review is CRD42023413715. We used PubMed, Google Scholar, ScienceDirect, and direct open-access journal (DOAJ) databases to find relevant studies. Initially, studies were exhaustively searched by using the full title ("Vaginal birth after cesarean section and its associated factors in Ethiopia") and keywords ("success rate," "successful," "vaginal birth after a cesarean section", "vaginal delivery after the cesarean section", "trial of labor after the cesarean section", "determinants", "associated factors", "predictors", "Ethiopia"). These keywords were connected using the Boolean operators ("OR" and/or "AND"). Besides this, reference lists of all included studies were assessed to find missed studies. Moreover, unpublished studies were searched in Ethiopian universities’ institutional repositories, mainly at the University of Gondar, Jimma, Addis Ababa, and Haramaya. The search details for PubMed were as follows: ("Successful" [All Fields] OR "Success rate" [All Fields]) AND ("vaginal birth after cesarean" [All Fields] OR "vaginal birth after cesarean" [MeSH Terms] OR ("vaginal"[All Fields] AND "birth"[All Fields] AND "after"[All Fields] AND "cesarean"[All Fields]) OR "vaginal birth after cesarean"[All Fields]) AND section[All Fields] AND ("determinants"[All Fields] OR "associated factors" [All fields] OR "Predictors" [All Fields]) AND ("Ethiopia"[MeSH Terms] OR "Ethiopia" [All Fields]). The search period was between 2005/01/01 and 2022/11/20 (Table S2).

### Eligibility criteria

The authors followed CoCoPop approaches (condition, context, and population) to establish search strategies and identify eligible studies.

*Inclusion criteria*: This systematic review and meta-analysis included articles that fulfilled the following criteria:

- *Condition (Co)*: We included studies that examined at least one or more of the following key outcomes: (1) success rate of vaginal birth after cesarean section; (2) determinants or associated factors of successful VBAC
- *Context (Co)*: We included studies that were conducted in Ethiopia.
- *Population (Pop)*: Studies that were done among laboring mothers with previous cesarean sections
- *Study design*: Cross-sectional and case–control studies
- *Publication condition*: Both published and unpublished studies
- *Language*: We included all studies written in the English language.

*Exclusion criteria*: We excluded studies that had a different outcome of interest.

### Outcome measurement

This meta-analysis study has two outcomes, namely, the success rate of vaginal birth after cesarean section and its associated factors.

*Station*: It refers to the relationship of the fetal head's lowermost portion in the pelvic canal with the ischial spines or indicates the degree of engagement of the presenting part. The station above the ischial spine was categorized as high (0), and the station below the ischial spine was categorized as low (> 0) in the pelvic examination^24^.

*Heterogeneity*: We used the following cut points to define the level of heterogeneity: When I-squared (I^2^) is zero, there is no heterogeneity; if the value is 25%, there is mild heterogeneity; if the value is 50%, there is moderate heterogeneity; if the value is 75%, there is high heterogeneity; and if the value is 100%, there is substantial heterogeneity.

#### Study selection, quality assessment, and data extraction process

All retrieved studies were exported to Endnote Version 7 software for screening. After the removal of duplicate studies, the remaining studies were evaluated for their relevance, accessibility of full text, outcomes of interest, and quality score. Finally, those studies that fulfilled the inclusion criteria and had high-quality scores were included.

The Newcastle–Ottawa quality assessment tool adapted for cross-sectional and case–control studies was used to assess the quality of the studies^28^. Two authors (DA and YB) assessed the quality of the studies individually. Disparities at the time of quality assessment were resolved through discussion and consensus by involving the third reviewer (NG). Finally, articles that received a score of 7 points out of 10 possible points for cross-sectional and case–control studies were considered high-quality and included in this study (Table S3).

Regarding data extraction, all the necessary data were extracted by two reviewers (DA and SB) using Microsoft Excel. The Excel contains the following components: The first author’s name, publication year, study region, study setting, study period, sample size, the success rate of VBA, and an adjusted odds ratio (AOR) with a 95% confidence interval for significant risk factors of successful VBAC.

### Statistical analysis

The data were analyzed using Stata version 17. A random effects model was selected to determine the pooled success rate of vaginal birth after a cesarean section and its associated factors^27–29^. Subgroup analysis and sensitivity analysis were conducted to identify the source of heterogeneity. Finally, publication bias was assessed by using Egger’s regression test^30^.

## Results

### Search results

**Figure 1:**
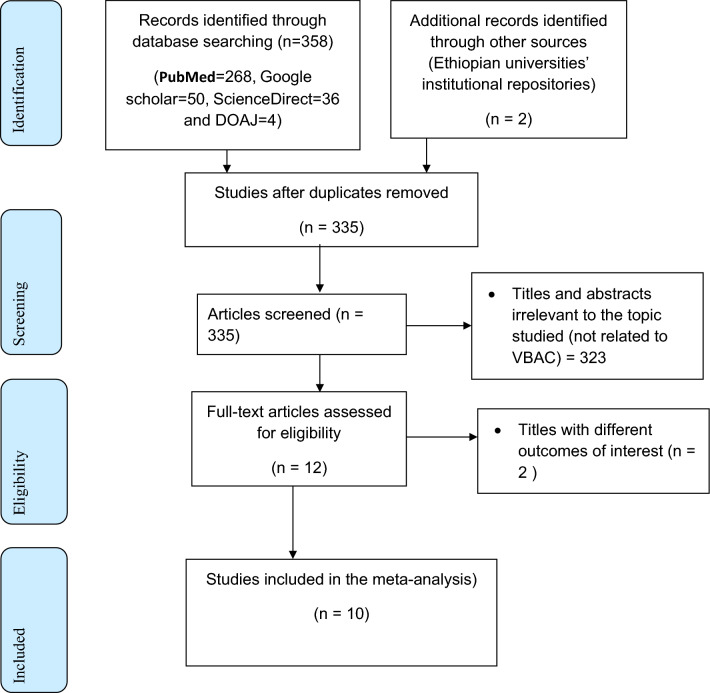
Flow chart describing study selection for systematic review and meta-analysis of the vaginal birth after cesarean section and its associated factors in Ethiopia.

A total of 360 studies were searched from different international databases and Ethiopian universities' institutional repositories. All the retrieved studies were transferred to Endnote 7 reference manager for screening. Then a total of 325 studies were removed due to irrelevant articles, duplication, and different outcomes of interest. Lastly, 10 studies that fulfilled the inclusion criteria were included (Fig. 1).

### Characteristics of included studies

This meta-analysis included a total of 10 studies with 2588 study participants^24,25,31–37^. Among these, five were cross-sectional studies^24,25,34,36^ and the remaining were case–control studies^31–33,35,37^. Concerning geographical distribution, four regions and one administrative City, namely, the Oromia region, the South Nation Nationalities and Peoples Region (SNNPR), the Amhara region, the Harar region, and Addis Ababa administrative City, were represented (Table 1).

**Table 1:** Descriptive summary of 10 studies reporting successful vaginal birth after cesarean section and its associated factors in Ethiopia.

Author	Publication year	Region	Study area	Data collection periods	Study design	Sample size	Success rate (%)
Derebe et al	2022	Amhara	Bahir Dar	January 1 to December 31, 2020	cross-sectional	345	35.07
Girma et al	2021	SNNPR	Mizan Tape	January to February 2020	cross-sectional	416	41
Misgan et al	2020	Addis Ababa	Addis Ababa	April 2015 to January 2016	cross-sectional	268	69.4
Birara et al	2013	Addis Ababa	Addis Ababa	May 2009 to May 2010	case–control	204	N/A
Mekonen et al	2021	Oromia	Ambo	June 1 to July 1, 2020	case–control	295	N/A
Girma et al	2020	Oromia	Asella	March 1 to 30, 2018	case–control	288	N/A
Tefera et al	2021	Harari	Harari and Dire Dewa	June to October 2020	case–control	220	N/A
Dereje et al	2022	Oromia	Wollega	February 29 to June 30, 2020	case–control	230	N/A
Kumbi et al	2014	Oromia	Jimma	January 1 to December 31, 2013	cross-sectional	153	52.3
Siraneh et al	2018	SNNPR	Gurage	October 01/2015 to September 30/2016	cross-sectional	169	44.5

*N/A* Not applicable (the study did not assess the success rate of VBAC).

### The pooled success rate of vaginal birth after cesarean section in Ethiopia

**Figure 2:**
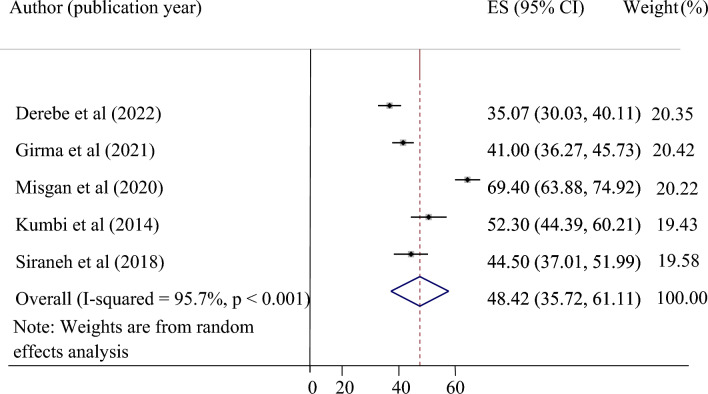
Forest plot showing the pooled success rate of VBAC in Ethiopia.

Five primary studies were included to determine the pooled success rate of vaginal birth after a cesarean section^24,25,34,36,38^. The pooled success rate of vaginal birth after a cesarean section was 48.42 with a 95% CI of 35.72 to 61.1. A marked type of heterogeneity was detected across the studies (I^2^ = 95.7%). The highest rate of successful vaginal birth after a cesarean section was reported by Misgan et al.^25^, while the lowest success rate of VBAC was reported by Derebe et al.^24^ (Fig. 2).

### Subgroup analysis

Subgroup analysis was done to identify the source of heterogeneity and minimize the random variations between the point estimates of primary studies and pooled success rate of VBAC by using sample size, publication status, and study period. However, heterogeneity was still observed between the studies. The overall success rate of VBAC was found to be 54.22% in published studies, 55.57% in studies with a sample size of less than 300, and 48.45% in studies conducted after 2018 (Table 2).

**Table 2:** Subgroup analysis of success rate of vaginal delivery after cesarean section in Ethiopia (n = 5).

Subgroup	Number of studies	Success rate (95%CI)	I^2^ and P-value
Publication status			
Published	3	54.22 (35.80,72.64)	(95.7%, p ≤ 0.001)
Unpublished	2	39.36 (30.16,48.56)	(76.1%, p = 0.041)
Sample size			
≥ 300	2	38.10 (32.29, 43.91)	(64.7%, p = 0.092)
< 300	3	55.57 (39.90, 71.25)	(93.5%, p ≤ 0.001)
Study period			
Before or at 2018	2	48.29 (40.65, 55.93)	(49.2%, p = 0.161)
After 2018	3	48.45 (28.65, 68.26)	(97.8%, p ≤ 0.001)

### Sensitivity analysis

Sensitivity analysis was done to check the influences of individual studies on the overall success rate of VBAC. There was no significant influence of individual studies on the pooled success rate of VBAC. When Derebe et al. and Misgan et al. were excluded from the analysis, the pooled success rate of VBAC was found to be high and low, with success rates of 51.82% and 42.67%, respectively (Table 3).

**Table 3:** A sensitivity analysis of the success rate of VBAC in Ethiopia when studies are omitted from the analysis.

Study omitted	Estimate	[95% Conf. Interval]
Derebe et al. (2022)	51.82	[37.65, 65.99]
Girma et al. (2021)	50.31	[33.68, 66.95]
Misgan et al. (2020)	42.67	[36.11, 49.23]
Kumbi et al. (2014)	47.48	[32.19, 62.76]
Siraneh et al. (2018)	49.38	[33.84, 64.92]
**Combined**	48.41	[35.71, 61.11]

### Publication bias

Publication biases were assessed objectively by using Egger’s regression test, and the results indicated that there was no publication bias or small study effect between the studies (p-value = 0.683).

### Factors associated with successful vaginal delivery after cesarean section

A total of nine studies reported one or more associated factors for successful VBAC. The most common factors reported by the majority of studies were a history of vaginal birth, a ruptured amniotic membrane at admission, and a cervical dilatation of four or more centimeters at admission (Table 4).

**Table 4:** Summary of factors associated with successful VBAC for each study.

Author	Publication year	Factors associated with successful VBAC	AOR	95% CI
Derebe et al	2022	Age < 30 years	2.71	1.21, 6.08
		Previous vaginal delivery	4.18	2.01, 8.71
		Cervical dilatation ≥ 4 cm at admission	2.2	1.06, 4.56
		Low station at admission	2.77	1.32, 5.81
		Ruptured membrane at admission	4.18	2.01, 8.71
Girma et al	2021	Cervical dilatation ≥ 4 cm at admission	2.7	1.47, 4.95
		Previous vaginal delivery	4	2.05, 7.83
Misgan et al	2020	Cervical dilatation ≥ 4 cm at admission	10	4.00, 25
		Ruptured membrane at admission	2.67	1.28, 5.57
Birara et al	2013	Cervical dilatation ≥ 4 cm at admission	6.63	3.36, 13.01
		No history of stillbirth	2.54	1.03, 6.24
		Low station at admission	10	4.00, 25
Mekonen et al	2021	Age < 30 years	5.37	2.28, 12.66
		Cervical dilatation ≥ 4 cm at admission	2.05	1.14, 3.67
		Previous vaginal delivery	3.85	1.84, 8.05
Girma et al	2020	Previous vaginal delivery	3.71	1.91, 7.25
		Ruptured membrane at admission	2.34	1.28, 4.28
Tefera et al	2021	No history of stillbirth	14.28	1.86, 25
		Low station at admission	5.26	1.04, 25
Dereje et al	2022	No history of stillbirth	4.2	1.20, 14.62
		Previous vaginal delivery	2.4	1.20, 6.40
Siraneh et al	2018	Cervical dilatation ≥ 4 cm at admission	8.17	3.3, 34.47

*AOR* Adjusted Odds Ratio, *CI* Confidence interval.

**Figure 3:**
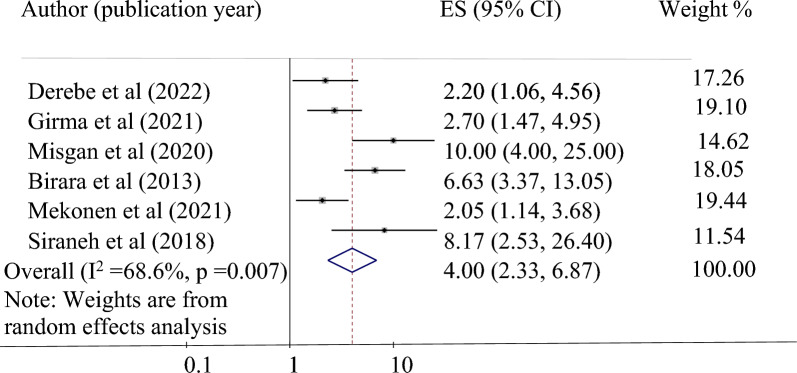
Forest plot for the association of a cervical dilation of 4 or more cm at admission with successful VBAC in Ethiopia.

*The relationship between cervical dilation at admission and vaginal birth after cesarean section*: The effect of cervical dilation at admission on successful vaginal birth after a cesarean section was evaluated using six studies^24,25,31,34–36^. In this study, cervical dilation of more than or equal to 4 cm at admission was found to be significantly associated with a successful vaginal birth after a cesarean section. Those mothers with cervical dilation ≥ 4 cm at admission were four times more likely to have a successful VBAC (pooled odds ratio 4, 95% CI 2.33, 6.8) (Fig. 3).

**Figure 4:**
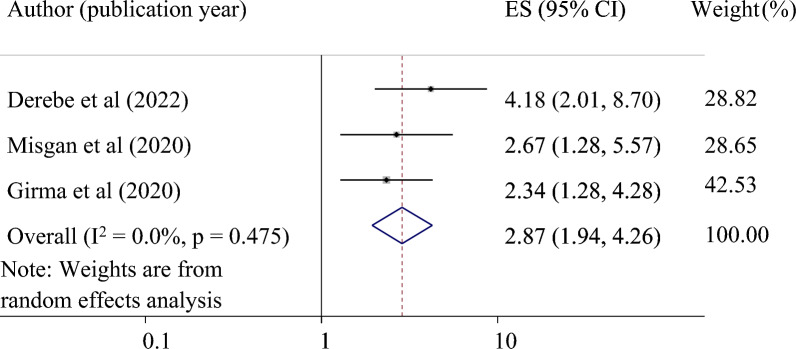
Forest plot for the association of ruptured amniotic membrane at admission with successful VBAC in Ethiopia.

*The association between ruptured amniotic membrane at admission and VBAC*: Three primary studies were used to determine the relationship between ruptured membranes and successful VBAC^24,25,33^. The result revealed that ruptured amniotic membranes at admission were significantly associated with successful VBAC. Those mothers who had a rupture of the amniotic membrane at admission were 2.87 times more likely to have a successful VBAC (pooled odds ratio 2.87, 95% CI 1.94, 4.26) (Fig. 4).

**Figure 5:**
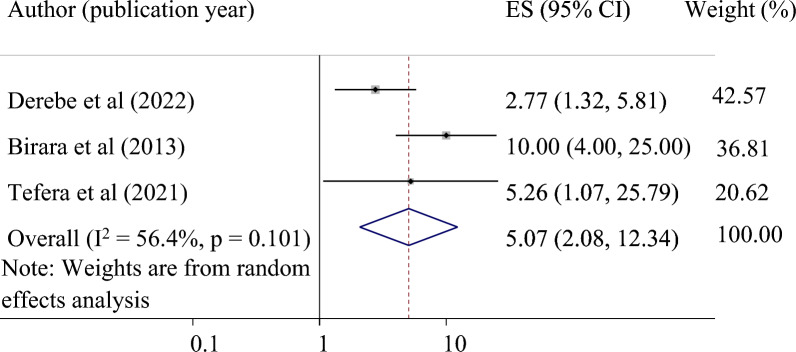
Forest plot showing the association between having a low station at admission and successful VBAC.

*The association between having a low station at admission and successful VBAC*: The association between having a low station at admission (station ≥ 0) and successful VBAC was examined using three studies^24,31,37^. The result indicated that having a low station at admission was significantly associated with a successful VBAC. Mothers who had a low station at admission were 5.11 times more likely to have a successful VBAC than mothers with a high station (pooled odds ratio 5.07, 95% CI 2.08, 12.34) (Fig. 5).

**Figure 6:**
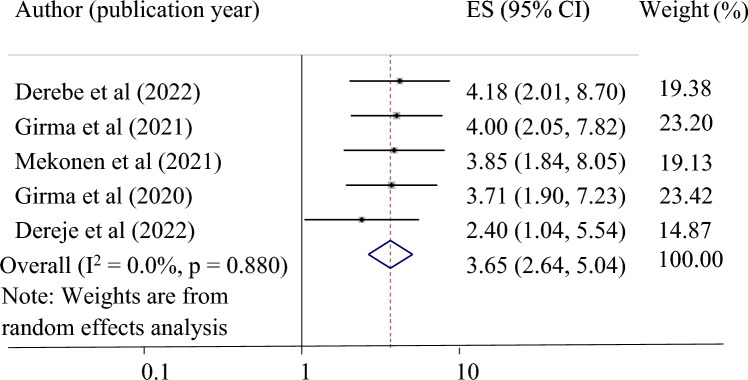
Forest plot showing the association between previous history of vaginal delivery and successful VBAC.

*The association between previous history of vaginal delivery and successful VBAC*: Five studies were used to investigate the relationship between previous vaginal delivery history and successful VBAC^24,32–35^. This study discovered that a previous history of vaginal birth was significantly associated with a successful VBAC. Those mothers with a previous history of vaginal delivery were 3.65 times more likely to have a successful vaginal birth after a cesarean section (pooled odds ratio 3.65, 95% CI 2.64, 504) (Fig. 6).

**Figure 7:**
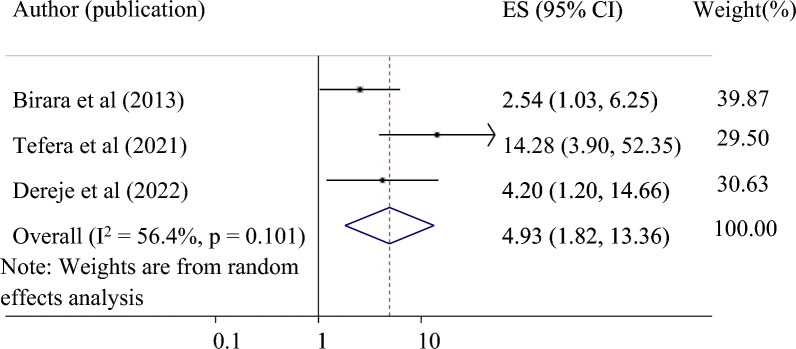
Forest plot for the association of no history of stillbirth with successful VBAC among women who had trial of labor after cesarean section in Ethiopia.

*The association between no history of stillbirth and successful VBAC*: Three primary studies were used to examine the relationship between no history of stillbirth and successful VBAC^31,32,37^. The result showed that mothers with no history of stillbirth were 4.93 times more likely to have a successful VBAC than mothers with a history of stillbirth (pooled odds ratio 4.93, 95% CI 1.82, 13.36) (Fig. 7).

**Figure 8:**
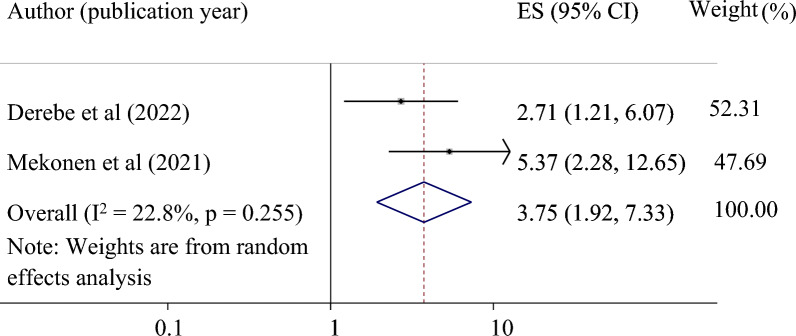
Forest plot for the association of maternal ages less than 30 years with successful VBAC in Ethiopia.

*The association between maternal age and successful VBAC*: Finally, we used two studies to examine the relationship between maternal age below 30 years and successful VBAC^24,35^. This study found that maternal age under 30 years was significantly associated with successful VBAC. Those mothers whose age was less than 30 years were 3.75 times more likely to have a successful VBAC as compared to those with older age (pooled odds ratio 3.75, 95% CI 1.92, 7.33) (Fig. 8).

## Discussion

This meta-analysis assessed the pooled success rate of VBAC and its associated factors in Ethiopia. To the best of our knowledge, this research is the first of its type in Ethiopia to examine the overall success rate of VBAC and the contributing factors.

The pooled success rate of VBAC was found to be 48.42% with a 95% CI of 35.72 to 61.1. This finding was lower than a study finding in Australia (64.4%)^39^, the UK (63.4%)^20^, and China (84%)^22^. This finding was also lower than a finding of a meta-analysis study in developed countries^40^. The variation in the success rate of VBAC across countries could be due to differences in hospital settings or eligibility criteria for a trial of labor after a cesarean section. Furthermore, the higher success rate of VBAC in the previous studies might be due to the availability of advanced labor monitoring machines, which might decrease the unnecessary repeated CS. In addition, the discrepancies might be due to the variations in the management modalities for labor abnormalities among those mothers who had TOLAC. In our country, CS is the only treatment option for prolonged labor secondary to poor uterine contractions among those mothers who had a TOLAC.

The finding of this study was also lower than the finding of a meta-analysis study in Sub-Saharan African countries, which has a 69% success rate of VBAC^41^. The variation in the success rate of VBAC might be due to disparities in sample size, where the previous study used several primary studies and a large sample size as compared to our study. In addition, discrepancies in the threshold level for TOLAC, intrapartum fetal monitoring, and quality of health services between the countries might contribute to this difference.

The finding of this study revealed that having a ruptured amniotic membrane at admission was significantly associated with successful VBAC. This finding was supported by a study finding in China^22^. This could be explained by the release of natural prostaglandins during the rupture of the amniotic membrane. Prostaglandin facilitates the progress of labor and decreases the chance of labor abnormalities, mainly poor uterine contractions^42^.

This study also found a strong association between previous history of vaginal delivery and successful VBAC. This finding is in agreement with a study finding in China^22^, Turkey^23^, and Thailand^14^. This finding is also in agreement with a meta-analysis study, which revealed that the history of previous vaginal deliveries is one of the most important factors for the success of VBAC^40^. The possible reason could be due to good psychological readiness and awareness of the advantage of vaginal delivery in those mothers with a history of vaginal delivery.

This study also found a strong association between a cervical dilatation of four centimeters or more at admission and a successful VBAC. Evidence from Pakistan^13^ and Nigeria^6^ supports the current finding. This might be because obstetric care providers usually encourage TOLAC when cervical dilatation is more than or equal to 4 cm. Furthermore, those mothers who are in the active phase of labor might have better progress of labor that results in successful VBAC as compared to the latent first stage of labor.

Maternal age below 30 years was also significantly associated with successful VBAC. Evidence from the meta-analysis study supports the present finding and revealed that younger women, especially those 35 years old, are more likely to have a successful and safe VBAC^40^. Another study also reported that maternal age of above 30 years was independently associated with Failed TOLAC^43^. This may be because older mothers are less likely to attempt TOLAC due to a fear of urine rupture.

Furthermore, we found a strong association between a low station at admission and a successful VBAC. This finding was supported by a previous study that reported that women who had a station lower than − 1 were significantly associated with successful VBAC^44,45^.

Lastly, no history of stillbirth was significantly associated with a successful VBAC. Evidence revealed that a history of fetal complications or adverse birth outcomes in the previous pregnancy increased the cesarean delivery rate^46^. Previous history of stillbirth during labor could influence women's preferences on the mode of delivery and the obstetric care provider's decision to halt or continue a vaginal birth in women with a history of cesarean section^1^.

This study has some limitations. The lack of studies from some regions might affect the generalizability of this study. Furthermore, the presence of heterogeneity across the studies might affect the pooled success rate of VBAC.

## Conclusion

The overall success rate of VBAC was low in Ethiopia. A successful VBAC was significantly associated with a history of vaginal birth, a ruptured amniotic membrane at admission, a low station at admission, age less than 30 years, cervical dilatation of four or more centimeters at admission, and no history of stillbirth. Therefore, the Ministry of Health should consider those identified factors and revise the management guidelines and eligibility criteria for TOLAC. Moreover, obstetric care providers should prevent repeated CS by providing appropriate antenatal counseling regarding influencing factors for VBAC and the chance of achieving a successful VBAC.

## Supplementary Information

Supplementary Information 1.

Supplementary Information 2.

Supplementary Information 3.

## Data Availability

The authors confirm that the data supporting the findings of this study are available within the article [and/or] its supplementary materials. Furthermore, the corresponding author (DA) will be contacted if someone wants to request the data from this study.

## Abbreviations

CS: Cesarean section
ES: Effect size
OR: Odds ratio
SNNPR: Southern Nations, Nationalities, and Peoples Region
TOLAC: Trial of Labor after Cesarean section
VBAC: Vaginal Birth after Cesarean section
