# Supplementary material for: Vaginal birth after cesarean section and its associated factors in Ethiopia: a systematic review and meta-analysis
Source: Sci Rep. 2023 May 15;13:7882. doi: 10.1038/s41598-023-34856-8 (PMC10185669; doi:10.1038/s41598-023-34856-8)
Supplement: Supplementary file 2 — Supplementary Information 2. [file 41598_2023_34856_MOESM2_ESM.docx]

**S2 Table:** Searching strategies for some databases to assess the pooled success rate of vaginal birth after cesarean section and its determinants in Ethiopia

| **Databases** | **Searching terms** | **Number of studies** | **Searching period** |
| --- | --- | --- | --- |
| PubMed/ MEDLINE | ("Successful" [All Fields] OR "Success rate" [All Fields]) AND ("vaginal birth after caesarean"[All Fields] OR "vaginal birth after cesarean"[MeSH Terms] OR ("vaginal"[All Fields] AND "birth"[All Fields] AND "after"[All Fields] AND "cesarean"[All Fields]) OR "vaginal birth after cesarean"[All Fields]) AND section[All Fields] AND ("determinants"[All Fields] OR "associated factors"[All fields] OR "Predictors"[All fields]) AND ("ethiopia"[MeSH Terms] OR "ethiopia"[All Fields]) | 268 | From 2005/01/01 to 2022/11/20 |
| Google scholar | “level” or "proportion" or "magnitude" AND ‘’successful’’ OR ‘’success’’ AND ‘’vaginal birth after cesarean section’’ OR "vaginal birth” and “after cesarean section" AND "determinants" OR "associated factors" or "predictors” and “Ethiopia” | 50 |  |
| ScienceDirect | success rate or successful and Vaginal birth after cesarean section or Vaginal delivery after cesarean section and associated factors or determinant factors and Ethiopia | 36 |  |
| DOAJ | vaginal birth after caesarean section and associated factors and Ethiopia | 4 |  |
| Gray literature (specifically unpublished studies) |  | 2 |  |
| Total searched articles |  | 360 |  |
| Finally, fulfill the eligibility criteria for our review |  | 10 |  |
