# Supplementary material for: Vaginal birth after cesarean section and its associated factors in Ethiopia: a systematic review and meta-analysis
Source: Sci Rep. 2023 May 15;13:7882. doi: 10.1038/s41598-023-34856-8 (PMC10185669; doi:10.1038/s41598-023-34856-8)
Supplement: Supplementary file 3 — Supplementary Information 3. [file 41598_2023_34856_MOESM3_ESM.docx]

**S3 Table: Quality of assessment of articles using Newcastle - Ottawa quality assessment Scale (NOS): (Adapted for cross-sectional studies)**

| Studies | **Selection** | | | | **Comparability** | **Outcome** | | **Total score** | **Quality of the study** |
| --- | --- | --- | --- | --- | --- | --- | --- | --- | --- |
|  | Representativeness  (1) | Sample size  (1) | Non-respondents  (1) | Ascertainment of the exposure (risk factor)  (2) | The subjects in different outcome groups are comparable, based on the study design or analysis. Confounding factors are controlled (2) | Assessment of the outcome  (2) | Statistical test  (1) |  |  |
| Derebe et al | * |  | * | ** | * | ** | * | 8 | High quality |
| Girma et al | * | * | * | * | * | ** | * | 8 | High quality |
| Misgan et al | * | * | * | * | ** | ** | * | 9 | High quality |
| kumbi et al | * |  | * | ** | * | ** | * | 8 | High quality |
| Siraneh et al | * | * | * | * | * | ** | * | 8 | High quality |

**Descriptions of quality measurement adapted for cross sectional study**

**Selection: (Maximum 5 stars or 5 points)**

1) Representativeness of the sample:

1. Truly representative of the average in the target population. * (all subjects or random sampling): **1 point**
2. Somewhat representative of the average in the target population. * (nonrandom sampling) : **1 point**
3. Selected group of users: **0**
4. d) No description of the sampling strategy: 0

2) Sample size:

1. Justified and satisfactory: **1 point**
2. Not justified: **0**

3) Non-respondents:

1. Comparability between respondents and non-respondents characteristics is established, and the response rate is satisfactory: **1 point**
2. The response rate is unsatisfactory, or the comparability between respondents and non-respondents is unsatisfactory: **0**
3. No description of the response rate or the characteristics of the responders and the non-responders: **0**

4) Ascertainment of the exposure (risk factor):

1. Validated measurement tool : **(2points)**
2. Non-validated measurement tool, but the tool is available or described: **(1 point)**
3. No description of the measurement tool. **0**

**Comparability: (Maximum 2 stars or 2 points)**

1) The subjects in different outcome groups are comparable, based on the study design or analysis. Confounding factors are controlled.

1. The study controls for the most important factor (select one): 1 point
2. The study control for any additional factor: 1 point

**Outcome: (Maximum 3 stars or points)**

1) Assessment of the outcome:

1. Independent blind assessment: **2 points**
2. Record linkage: **2 points**
3. Self-report: **1 point**
4. No description: **0**

2) Statistical test:

1. The statistical test used to analyse the data is clearly described and appropriate, and the measurement of the association is presented, including confidence intervals and the probability level (p value): **1 point**
2. The statistical test is not appropriate, not described or incomplete. **0**

**Note: 1 asterisk or star (*) is equivalent to 1 point**

**Decisions of on the quality of the studies were based on the sum or total score:**

- **High quality studies: 7-10 points**
- **Low quality studies: 0-6 points**

**S3 Table: Quality of assessment of articles using Newcastle - Ottawa quality assessment Scale (NOS) Adapted for case control studies**

| Studies | **Selection** | | | | **Comparability** | **Exposure** | | | **Total score** | **Quality of the study** |
| --- | --- | --- | --- | --- | --- | --- | --- | --- | --- | --- |
|  | Representativeness of the cases  (1) | Is the case definition adequate  (1) | Selection of Controls  (1) | Definition of Controls (1) | Comparability of cases and controls on the basis of the design or analysis (2) | Ascertainment of exposure  (2) | Same method of ascertainment for cases and controls  (1) | Non-Response rate (1) |  |  |
| Birara et al | * | * | * | * | ** | ** | * | * | 10 | High quality |
| Mekonen et al | * | * | * | * | ** | ** | * | * | 10 | High quality |
| Girma et al | * | * | * | * | ** | ** | * | * | 10 | High quality |
| Tefera et al | * | * | * | * | ** | ** | * | * | 10 | High quality |
| Dereje et al | * | * | * | * | ** | ** | * | * | 10 | High quality |

**Descriptions of quality measurement adapted for case control studies**

**Selection: (Maximum 4 stars or 4 points)**

1. **Is the Case Definition Adequate?**
2. Requires some independent validation (e.g. >1 person/record/time/process to extract information, or reference to primary record source such as x-rays or medical/hospital records)
3. Record linkage (e.g. ICD codes in database) or self-report with no reference to primary record
4. No description
5. **Representativeness of the Cases**
6. All eligible cases with outcome of interest over a defined period of time, all cases in a defined catchment area, all cases in a defined hospital or clinic, group of hospitals, health maintenance organisation, or an appropriate sample of those cases (e.g. random sample)
7. Not satisfying requirements in part (a), or not stated.
8. **Selection of Controls**

This item assesses whether the control series used in the study is derived from the same population as the cases and essentially would have been cases had the outcome been present.

1. Community controls (i.e. same community as cases and would be cases if had outcome)
2. Hospital controls, within same community as cases (i.e. not another city) but derived from a hospitalised population
3. No description
4. **Definition of Controls**
5. If cases are first occurrence of outcome, then it must explicitly state that controls have no history of this outcome. If cases have new (not necessarily first) occurrence of outcome, then controls with previous occurrences of outcome of interest should not be excluded.
6. No mention of history of outcome

***COMPARABILITY (maximum 2 stars or points)***

- Either cases or controls must be matched in the design and/or confounders must be adjusted for in the analysis: **
- Statements of no differences between groups or that differences were not statistically significant are not sufficient for establishing comparability.
- Note: If the odds ratio for the exposure of interest is adjusted for the confounders listed, then the groups will be considered to be comparable on each variable used in the adjustment.

***EXPOSURE (maximum 4 stars or points)***

1. Ascertainment of Exposure (maximum 2 points or stars)
2. Non-Response Rate ( 1 star or point)
3. Same method of ascertainment for cases and controls (1 star or point)

**Note: 1 asterisk or star (*) is equivalent to 1 point**

**Decisions of on the quality of the studies were based on the sum or total score:**

- **High quality studies: 7-10 points**
- **Low quality studies: 0-6 points**
